# What is the impact of underweight on self-reported health trajectories and mortality rates: a cohort study

**DOI:** 10.1186/s12955-017-0766-x

**Published:** 2017-10-02

**Authors:** Geir Fagerjord Lorem, Henrik Schirmer, Nina Emaus

**Affiliations:** 1 Department of Health and Care Sciences, Faculty of Health Sciences, UiT The Arctic University of Norway, Tromsø, Norway; 2 Department of Clinical Medicine, Faculty of Health Sciences, UiT The Arctic University of Norway, Tromsø, Norway; 3 Department of Clinical Medicine, Faculty of Medicine, University of Oslo, Oslo, Norway; 4 Division of Medicine, Akershus University Hospital, Lørenskog, Norway

**Keywords:** Body mass index, Mortality, Self-reported health, Thinness, The Tromsø study

## Abstract

**Background:**

Utilizing a cohort study design combining a survey approach with repeated physical examinations, we examined the independent effects of BMI on mortality and self-reported health (SRH) and whether these independent effects change as people grow older.

**Methods:**

The Tromsø Study consists of six surveys conducted in the municipality of Tromsø, Norway, with large representative samples of a general population. In total, 31,985 subjects participated in at least one of the four surveys administered between 1986 and 2008. Outcomes of interest were SRH and all-cause mortality.

**Results:**

Overweight and underweight subjects reported significantly lower levels of SRH, but age affected the thinnest subjects more than all others. The SRH trajectory of underweight subjects at age 25 was slightly above the other categories (0.08), but it fell to −.30 below the reference category at age 90. For obese subjects, the difference was −0.15 below the reference category at age 25 and −0.18 below at age 90. This implies that even though a low BMI was slightly beneficial at a young age, it represented an increasing risk with age that crossed the reference curve at age 38 and even crossed the obese trajectory at age 67 in the full fitted model. The proportional hazard ratio for those who were underweight was 1.69 (95% CI: 1.38-2.06) for all-cause death as compared to 1.12 (95% CI: 1.02-1.23) for obese subjects.

**Conclusion:**

BMI affected SRH and all-cause mortality independently from comorbidity, mental health, health-related behaviors and other biological risk factors. Being underweight was associated with excess mortality as compared to all others, and age affected the thinnest subjects more than all others. Weight increase was beneficial for mortality but not for SRH among the underweight. The rapid decline of SRH with increasing age suggests that particular attention should be paid to underweight after 38 years of age.

**Electronic supplementary material:**

The online version of this article (10.1186/s12955-017-0766-x) contains supplementary material, which is available to authorized users.

## General advice for clinicians

1. Low body mass is not beneficial in the long term; it affects self-reported health and is associated with excess mortality. It is crucial to avoid underweight, not only overweight. The golden mean is lower than the population mean but should not be too low.
2. Increasing weight to the normal range (18.5-25 kg/m^2^) from underweight (<18.5 kg/m^2^) is beneficial for comorbidity and mortality but not necessarily self-reported health.
3. There are more daily smokers among those with low BMI, and just as many physically inactive as among those who are overweight. General advice on health-related behavior is therefore also valid for those who are thin.
4. It is the sum of risks that is crucial, and underweight persons should be screened for risk to the same extent as obese persons

## Background

Being thin is an ideal embedded in Western culture [1], but is it healthy? Being thin or underweight (i.e. BMI <18.5 kg/m^2^) is not necessarily a negative characteristic [2–4]. It constitutes a rather complex group, since people can have a low BMI for a number of reasons: It can be the result of healthy living (e.g. healthy diet and exercise), but it may also apply to individuals with eating disorders or be due to malnutrition or weight loss due to clinical or sub-clinical disease [5–7].

Underweight can potentially by itself lead to adverse health effects such as higher disease burden, it affects the outcome for several medical conditions, and it is a known risk factor for older people (>65 years) [8–15]. Weight loss is another factor known to have a potential negative effect on health [16–20]. Even a modest decline in body weight is a known risk of mortality in older adults [21]. It has been shown how body weight declines three to 5 years before cardiovascular deaths [22]. It is known that Individuals with eating disorders have significantly elevated mortality rates [23], and these conditions have a severe impact on the patient’s health-related quality of life [24]. However, it remains unanswered how these outcomes are affected by low BMI in a general population and whether this risk changes when people grow older.

Our intention was to examine how body mass affected two different aspects of health: Mortality, which concerns survival, and self-rated health (SRH), which is a person’s general assessment of his/her own health and health-related quality of life. SRH is associated with a broad range of objective health outcomes, including mortality [25], subclinical and clinical disease [26], and health service use [27]. Since being thin can also be beneficial for health, it is of interest to investigate whether the SRH benefits at some point in life outweigh the disadvantages of very low body mass. We thus wanted to examine the relationship between underweight (i.e. BMI <18.5 kg/m^2^), SRH and mortality.

## Methods

Our aim was to estimate the independent effects of BMI on mortality and SRH from youth and estimate whether any independent effects change as people grow older. The design and comprehensive database of the Tromsø Study allowed us to examine the relationship between body weight and SRH throughout life in the general population, including the impact of a broad range of other health-related factors. The Tromsø Study (TS) consisted of six repeated population health examinations carried out in 1974 (TS1), 1979-80 (TS2), 1986-87 (TS3), 1994-95 (TS4), 2001 (TS5), and 2007-08 (TS6). The six surveys had the same general design. Based on the official population registry, residents of the municipality of Tromsø were invited to take part in the survey. The aim was to include large, representative samples of the Tromsø population, with the invitation of entire birth cohorts and random samples. The attendance rate was high (66-75%). In all surveys, a questionnaire was enclosed in the invitation. Over the years, the questionnaires were expanded to include questions about a wide range of diseases and symptoms, dietary habits, other lifestyle aspects, use of medication, sleeping patterns, socio-economic status, and use of health care services. The physical examination consisted of blood samples and measurements of blood pressure, height and weight [28]. SRH was introduced in 1986.

### Data

We used TS 3-6 for the latent trajectory model. In total, 31,985 subjects participated in at least one of the four surveys administered between 1986 and 2008. Three thousand six hundred forty-nine subjects participated each time, 5905 thrice, 8664 twice, while 13,766 subjects participated once. The latent trajectory model required at least two measuring points and thus included 9506 men and 8712 women [29].

We used TS4 with updated measures for the Cox proportional hazard model. TS4 was executed in 1994-95 and included 12,014 men and 13,237 women aged 25 to 97 at baseline. We excluded subjects who had missing values for SRH (*n* = 40) or missing medical conditions, physical condition or mental health symptoms (*n* = 886). Subjects who attended in 2001 (*n* = 6814) and 2007/8 (*n* = 9316) had their SRH and risk factor values updated at the time of their examination. Follow-up time extended from the date of study entry in 1994 to the date of death or the end of study follow-up on December 31, 2015.

### Variables

The participants completed well-validated questionnaires that included questions on a broad range of diseases, symptoms, health behavior, social conditions, education, and level of physical activity [28]. Outcomes of interest were SRH and all-cause mortality. SRH was reported by answering the survey question ‘What is your current state of health?’ in a range from Poor (1) to Very Good (4). Time of death was retrieved from the Norwegian National Causes of Death Registry. The degree of coverage of the registry is near-complete [30].

**Fig. 1:**
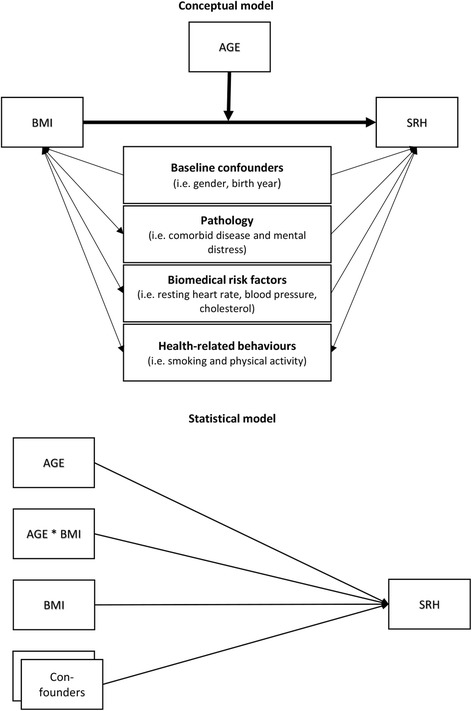
Directed acyclic graph showing the causal relationships that guide the conceptual model and its transition into the statistical model

BMI and age were the independent variables of interest. The conceptual model was that BMI has an effect on SRH that is moderated by age (Fig. 1). The other variables were treated as confounders for the sake of the presentation. Age was registered at attendance. Specially trained personnel took non-fasting blood samples and measured blood pressure (SBP/DBP), resting heart rate (RHR), body weight and height. Comorbid diseases were self-reported specific medical conditions. We selected 13 symptomatic medical conditions reported in all panels. These were psoriasis, food allergies, chronic bronchitis, migraine, ulcer, asthma, thyroid disease, arthritis, myocardial infarction, cerebrovascular stroke, diabetes, osteoporosis, and angina, which all have a varied impact on SRH. We therefore utilized the Health Impact Index (HII) to measure the comorbid conditions. HII classifies patients with comorbid disease according to the impact that each condition has on SRH by assigning a weight to each condition. HII equals the total score of each condition of the participant. HII thus considers both the severity and joint effects of the conditions [31].

Mental distress was based on well validated self-report symptom inventories comprising questions representative of the symptom configurations of anxiety and depression commonly observed among outpatients. Each answer is scored from 1 to 4. The measurement is the average score. The mental health index (CONOR-MHI) was used at TS4. In the following surveys (TS5-6), the Hopkins Symptom Checklist (HSCL) was used [32]. The CONOR-MHI has been compared with the HSCL with reasonably good agreement. It was highly correlated with HSCL-10 (*r* = 0.8). A cut-off level of 2.15 for significant symptoms is equivalent to the 1.85 level in HSCL-10 [33]. We standardized the variable for better longitudinal comparison in the regression model. TS3 did not include any validated mental health measures and these were coded as missing.

Physical exercise in leisure time was measured by answers to ‘How often do you exercise?’, categorized as inactive (0), light (1), moderate (2-3), and intensive (3-7) physical activity. This scale has been widely used in Scandinavian studies, and physical activity levels have been correlated with physical fitness [34].

### Analysis

We performed the analysis in three stages using Stata v14. The first stage examined the overall distribution of SRH for age and BMI groups for each panel; the second stage utilized the repeated measures to estimate how SRH changed for different BMI groups as the subjects grow older. The ability of SRH to predict all-cause death is well-known [25]. The third stage thus examined whether BMI and SRH also affected mortality.

- Stage 1: We used cross tabulation and ANOVA to examine the distribution of the subjects and mean SRH for age versus BMI. We divided the subjects into 10-year age groups and BMI into <18.5 Kg/m^2^ (Underweight), 18.5-22.99 kg/m^2^ (Lower normal) 23-25.99 kg/m^2^ (Upper normal), 25-30 kg/m^2^, >30 kg/m^2^ (Obese). BMI >25 kg/m^2^ is also referred to as overweight [2–4]. We know that SRH declines with increasing age [31], and we used ANOVA to determine the effect of BMI on SRH for the age groups within each panel. The post hoc analysis included a pairwise comparison of the BMI groups.
- Stage 2: We used latent trajectory modeling to examine how SRH changes over time. The method is also known as growth curves because it was first used to estimate the normal growth rate of infants. It consists of multilevel regression modeling that utilizes the fact that the data are multiple observations over time nested within subjects. Latent trajectory models are a special case of random coefficient models where we allow the coefficient of time to vary randomly between subjects. This method allowed us to explicitly model the shape of trajectories of individual subjects over time and how these trajectories varied based on occasion- and subject-level covariates [29].

Our purpose was to investigate the relationship between SRH and age and how this relationship varied between different BMI groups. The units for a longitudinal setting are time of attendance (j) and the clusters are subjects (i). There are different ways of modeling nonlinear development, using polynomial functions. In our study, time is represented as age, cohort, and period. Age_ij_ is the age at the date of the survey for each subject. Cohort is the birth year. Period_i_ is the attendance year for each occasion. Time is described by the equation Age_ij_ = Period_i_ - Cohort_j_. One time variable will thus be collinear with the two others and can be left out of the model. We can now consider a model that includes the two time scales (Age_ij_ and Period_i_) as covariates as well as gender, pathology (comorbid diseases and mental distress), physical examination measurements (resting heart rate, BMI, cholesterol and systolic blood pressure), physical exercise and smoking habits. We started by modeling the time as linear, then quadratic, cubic and quartic. We also modeled interaction between all covariates with age. Interaction coefficients with *p* > .05 were removed from the model one at a time until we reached the final model:

$$ {\mathrm{SRH}}_{\mathrm{ij}}=\kern0.5em {\upbeta}_1{\mathrm{bmi}}_{\mathrm{ij}}+{\upbeta}_2\mathrm{bmi}\ast {\mathrm{age}}_{\mathrm{ij}}+{\upbeta}_3{\mathrm{age}}_{\mathrm{ij}}+{\upbeta}_4{\mathrm{age}}_{\mathrm{ij}}^2+{\upbeta}_5{\mathrm{age}}_{\mathrm{ij}}^3+{\upbeta}_6{\mathrm{age}}_{\mathrm{ij}}^4+{\upbeta}_7{\mathrm{period}}_{\mathrm{j}}+{\upbeta}_8{\mathrm{sex}}_{\mathrm{j}}+{\upbeta}_9{\mathrm{HII}}_{\mathrm{ij}}+{\upbeta}_{10}\mathrm{MHI}/{\mathrm{HSCL}}_{\mathrm{ij}}+{\upbeta}_{11}\mathrm{resting}\ {\mathrm{HR}}_{\mathrm{ij}}+{\upbeta}_{12}{\mathrm{bloodpressure}}_{\mathrm{ij}}+{\upbeta}_{13}{\mathrm{cholesterol}}_{\mathrm{ij}}+{\upbeta}_{14}\mathrm{Phys}.{\mathrm{act}}_{\mathrm{ij}}+{\upbeta}_{15}{\mathrm{smoke}}_{\mathrm{ij}}+{\upbeta}_0+{\upzeta}_{1\mathrm{j}}+{\upvarepsilon}_{\mathrm{ij}} $$

The random intercept (*ζ*_1*j*_) and the occasion specific error (*ε*_*ij*_) allow the responses (*SRH*_*ij*_) to deviate from the polynomial function. Adding age to the slope in the random part (*ζ*_2 *ageij*_) did not significantly improve the model. The figure is the response (*SRH*_*ij*_) as a function of age and BMI.

Loss to follow-up always causes loss of information that cannot be recovered. The concern is that loss to follow-up can be systematic due to known or underlying physical conditions. The simplest approach to dealing with missing data is to restrict the analysis to complete cases (CC), i.e. individuals with no missing values. This can induce bias; however, inverse probability weighting (IPW) is a commonly used method to correct such bias [35]. The probabilities are obtained by modeling the observed loss to follow-up as a function of subject characteristics that determined the group recorded at baseline. These were BMI, comorbidity, blood pressure, cholesterol, and resting heart rate. We found that thin subjects and those with higher levels of comorbidity, resting heart rate and blood pressure were more likely to be missing, while higher cholesterol levels were beneficial. Weights from the model ranged from 1.07 to 1.81, with a mean of 1.29. We then performed a CC analysis, but weighted the complete cases by the inverse of their probability of not being missing. That meant that those with a small chance of being observed were given increased weight, to compensate for similar subjects who were missing.

- Stage 3: We used Cox proportional hazard regression to estimate hazard ratios (HRs) of all-cause death using baseline scores for BMI and gender in addition to age, comorbid disease, mental distress, and physical examination scores as time-dependent covariates updated in 2001 and 2007-08. Time at risk was the person-time measured in days from the first attendance date. The proportional hazard assumption was verified for BMI by visual inspection of log minus log survival curves and by tests of Schoenfeld residuals; we therefore included the intereaction between time and SRH in the model.

## Results

### Characteristics of the sample

Table 1 shows the distribution of observations for each BMI group. Underweight subjects reported lower levels of SRH in all panels and also more comorbid conditions. Most underweight subjects are among the youngest (<30 years) and oldest subjects (>70 years) in all panels. The age-standardized proportion of underweight subjects declined from 2.8% in TS3 to 1.5% in TS4, 1.3% in TS5, and 0.8% in TS6. Most of the underweight subjects were females (~77-85%). Among the underweight, the proportion who smoke is greater and the proportion who exercise at least once a week is smaller. The cardiovascular risk factors are both against and in favor of the group. When comparing the underweight subjects with the obese, we notice lower resting heart rates, fewer smokers and slightly higher physical activity levels among the obese. Blood pressure and cholesterol levels were better among those who were underweight, whereas comorbidity and mental distress levels were better in the obesity group. The SRH levels were better for the underweight group in T3; in T4-6, however, the obese subjects had higher SRH than the underweight subjects.

**Table 1:** Characteristics of all participants for BMI groups for each wave

	Panel	<18.5	18.5-22.99	23-24.99	25-29.99	>30	*P*-value ^c^
		Kg/m^2^	Kg/m^2^	Kg/m^2^	kg/m^2^	kg/m^2^	
Frequency	Tromsø 3	299	7075	4588	5226	945	
	Tromsø 4	327	6958	5789	9391	2730	
	Tromsø 5	86	1378	1534	3392	1484	
	Tromsø 6	80	1996	2175	5355	2454	
SRH (% reporting good/very good)	Tromsø 3	75.4%	82.0%	82.4%	76.9%	68.3%	<0.001
	Tromsø 4	54.8%	74.5%	74.1%	68.7%	54.9%	<0.001
	Tromsø 5	41.3%	64.6%	68.5%	64.4%	54.0%	<0.001
	Tromsø 6	46.6%	70.2%	71.9%	67.0%	53.9%	<0.001
Age (Mean)^a^	Tromsø 3	35.3	37.2	40.0	42.5	43.1	<0.001
	Tromsø 4	49.5	44.1	46.7	50.1	53.2	<0.001
	Tromsø 5	64.5	56.7	58.5	60.7	61.9	<0.001
	Tromsø 6	63.4	56.5	57.4	59.0	59.1	<0.001
Gender (% male)	Tromsø 3	17.5%	35.6%	58.6%	67.5%	50.8%	<0.001
	Tromsø 4	22.3%	33.5%	51.5%	58.3%	42.3%	<0.001
	Tromsø 5	15.0%	31.1%	43.2%	50.6%	39.8%	<0.001
	Tromsø 6	23.3%	29.5%	46.4%	54.1%	47.2%	<0.001
Comorbidity (mean HII)^b^	Tromsø 3	0.54	0.43	0.44	0.54	0.70	<0.001
	Tromsø 4	1.26	0.78	0.82	1.04	1.39	<0.001
	Tromsø 5	2.50	1.54	1.38	1.67	2.27	<0.001
	Tromsø 6	2.53	1.44	1.40	1.57	2.11	<0.001
Mental distress (mean MHI/HSCL)^b^	Tromsø 3	.	.	.	.	.	
	Tromsø 4	1.66	1.54	1.51	1.50	1.54	<0.001
	Tromsø 5	1.32	1.30	1.24	1.22	1.25	<0.001
	Tromsø 6	1.43	1.31	1.28	1.27	1.31	<0.001
Resting heart rate (mean BPM)^b^	Tromsø 3	80.7	75.2	73.0	73.8	78.0	<0.001
	Tromsø 4	76.9	72.8	71.2	72.1	75.8	<0.001
	Tromsø 5	75.4	71.1	69.3	69.6	72.7	<0.001
	Tromsø 6	70.3	64.7	63.5	64.7	67.6	<0.001
Systolic blood pressure (mean mmHG)	Tromsø 3	120.3	123.4	128.1	133.4	138.1	<0.001
	Tromsø 4	129.5	127.5	133.1	139.4	148.2	<0.001
	Tromsø 5	137.3	132.4	135.7	140.6	144.6	<0.001
	Tromsø 6	132.3	129.0	132.3	138.3	142.1	<0.001
Total cholesterol (mean)	Tromsø 3	5.25	5.45	5.89	6.26	6.48	<0.001
	Tromsø 4	5.72	5.65	5.99	6.35	6.59	<0.001
	Tromsø 5	5.91	5.90	6.07	6.26	6.31	<0.001
	Tromsø 6	5.53	5.45	5.59	5.69	5.61	<0.001
Physical activity (% inactive)	Tromsø 3	8.4%	10.8%	10.0%	11.0%	9.2%	0.255
	Tromsø 4	64.3%	46.6%	45.1%	50.8%	62.7%	<0.001
	Tromsø 5	48.5%	29.6%	30.8%	32.7%	37.5%	<0.001
	Tromsø 6	31.7%	15.2%	17.1%	18.9%	30.3%	<0.001
Smoking habits (% daily smokers)	Tromsø 3	64.3%	50.4%	43.1%	41.0%	35.0%	<0.001
	Tromsø 4	59.7%	44.4%	36.4%	31.3%	26.7%	<0.001
	Tromsø 5	67.5%	41.2%	30.6%	24.7%	18.3%	<0.001
	Tromsø 6	37.5%	26.3%	22.3%	17.7%	15.9%	0.003

^a^Tromsø 3 and 4 did not include subjects <25 years, Tromsø 5 &6 did not include subjects <30 years

^b^ *HII* Health impact index which measures comorbidity, *MHI* Mental health index (Tromsø 4), *HSCL* Hopkins symptoms checklist (Tromsø 5-6), *BPM* beats per minute

^c^Comparison of BMI groups were performed utilizing Kruskal-Wallis equality-of-populations rank test for gender, smoking habits and physical activity levels, and one-way anova for all other variables

When we tracked those who were underweight in 1986 (*n* = 299), we see that 27.4% (*n* = 109) died during the 29 years of follow-up. The mortality rate was 0.021, which is twice the rate (0.010) of the lower normal weight category (18.5-23 kg/m^2^). This occurred in spite of 76% of them being younger than 40 and a gradual increase in the mean BMI from 18.1 to 20.9 kg/m^2^ (BMI_diff_ = 0.74, Range − 2.80, 3.52). Some subjects changed weight drastically: 16 became overweight (25-29.99 kg/m^2^), and 15 reached the upper normal weight range, but most subjects (*n* = 193) remained in or below the lower normal range (<23 kg/m^2^). Fifty-eight of those remained underweight, and 75 were observed only once in the dataset. Remaining in the initial weight group was beneficial for SRH and comorbidity (HII) for all BMI categories with one exception, viz. those who had been thin and ended up within the normal weight range (18.5-24.99 kg/m^2^) at the end of the study. This group reported significantly fewer comorbid conditions (0.91 vs. 2.22) and their mortality rate (.0023 deaths per person-year) was lower than for those who remained underweight throughout the study. Despite this, 68.4% reported good or very good SRH in contrast to 77.8% of those who remained thin (Additional file 1: Table S1).

**Fig. 2:**
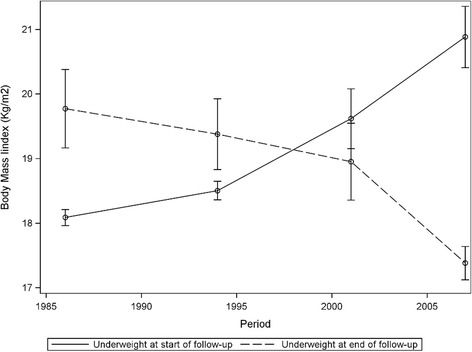
Change in body mass (BMI) for subjects who were underweight at beginning and end of follow-up period with 95% confidence intervals

Those who were the thinnest subjects at the final panel (*n* = 80), had on average lost weight (BMI_diff_ = −1.77, Range: −7.53, 0.38). Twenty had remained underweight, 45 had lost weight from 18.5-22.99 kg/m^2^, five subjects had been overweight and only one of those had been obese, while 10 were observed only once in the dataset. 25.3% (*n* = 20) died during the remaining 8 years of follow-up (mortality rate 0.031). Figure 2 shows how the BMI of those who started or ended as underweight in our study changed over the time of observation.

### The overall effect of BMI

Table 2 shows the observations according to age and BMI for each panel and the distribution of SRH across age and BMI. One-way ANOVA showed a significant effect of BMI on levels of SRH for almost all age groups in all cross-sectional panels. Pairwise comparison revealed that those who were overweight and underweight reported significantly lower SRH levels, while the differences within the upper and lower normal range often did not show significant differences. SRH was highest for the upper normal range (23-24.99 kg/m^2^) in all age groups except for two exceptions in the oldest age group, in which overweight subjects (25-29.99 kg/m^2^) had marginally but statistically significant higher levels of SRH in TS3 and TS6. The upper normal range is therefore used as reference category for the regression models. The youngest underweight subjects reported only slightly lower SRH levels than the reference category, while the underweight subjects at 70 years were below all other BMI categories.

**Table 2:** Cross section of Self-reported health for BMI and 10-year age groups for Tromsø 3-6

	<18,5 Kg/m^2^			18,5-22,99 Kg/m^2^			23-24,99 Kg/m^2^			25-29,99 Kg/m^2^			>30 Kg/m^2^			*p*-value
	Count	Mean	(SD.)	Count	Mean	(SD.)	Count	Mean	(SD.)	Count	Mean	(SD.)	Count	Mean	(SD.)	
Tromsø 3																
25-29	88	3.09	(0.64)	1529	3.24	(0.68)	692	3.29	(0.68)	483	3.20	(0.70)	86	3.05	(0.78)	0.008
30-39	138	3.00	(0.74)	31	3.13	(0.70)	1681	3.18	(0.70)	1624	3.06	(0.71)	250	2.80	(0.74)	<0.001
40-49	44	2.67	(0.79)	1626	3.00	(0.74)	1338	3.04	(0.73)	1779	2.95	(0.73)	331	2.85	(0.75)	<0.001
50-59	27	2.33	(0.87)	741	2.75	(0.81)	777	2.87	(0.79)	1166	2.81	(0.81)	248	2.58	(0.83)	<0.001
60-69	2	1.00		79	2.62	(0.82)	100	2.60	(0.81)	174	2.73	(0.77)	30	3.00	(0.65)	0.031
Tromsø 4																
25-29	51	2.94	(0.68)	993	3.18	(0.64)	600	3.21	(0.64)	685	3.13	(0.63)	147	2.90	(0.63)	<0.001
30-39	70	3.03	(0.59)	2138	3.12	(0.65)	1468	3.10	(0.64)	1784	3.04	(0.61)	396	2.85	(0.66)	<0.001
40-49	61	2.67	(0.86)	1853	2.92	(0.66)	1588	2.92	(0.64)	2485	2.89	(0.62)	646	2.70	(0.66)	<0.001
50-59	41	2.49	(0.75)	829	2.69	(0.70)	1011	2.78	(0.67)	1914	2.71	(0.65)	610	2.55	(0.66)	<0.001
60-69	41	2.17	(0.77)	599	2.50	(0.64)	615	2.52	(0.63)	1364	2.53	(0.63)	469	2.38	(0.62)	<0.001
70-79	46	1.96	(0.79)	408	2.40	(0.69)	387	2.45	(0.68)	925	2.42	(0.65)	372	2.28	(0.68)	<0.001
> 80	17	2.00	(0.61)	138	2.26	(0.68)	120	2.41	(0.69)	234	2.28	(0.60)	90	2.18	(0.61)	0.034
Tromsø 5																
30-39	8	2.75	(0.71)	167	3.14	(0.68)	135	3.13	(0.60)	177	3.05	(0.61)	73	2.99	(0.52)	0.163
40-49	4	2.75	(0.50)	323	3.00	(0.67)	300	3.07	(0.67)	547	2.99	(0.63)	178	2.82	(0.61)	0.002
50-59	7	2.71	(0.76)	156	2.66	(0.69)	197	2.78	(0.65)	481	2.71	(0.62)	237	2.59	(0.62)	0.038
60-69	29	2.34	(0.77)	369	2.64	(0.65)	502	2.71	(0.66)	1234	2.69	(0.63)	549	2.51	(0.59)	<0.001
70-79	35	2.21	(0.69)	311	2.46	(0.61)	358	2.59	(0.64)	846	2.54	(0.62)	392	2.40	(0.59)	<0.001
> 80	3	1.33	(0.58)	52	2.45	(0.68)	42	2.54	(0.60)	107	2.42	(0.66)	55	2.31	(0.60)	0.024
Tromsø 6																
30-39	2	3.00	(0.00)	111	3.04	(0.82)	93	3.04	(0.69)	166	2.96	(0.65)	85	2.71	(0.72)	0.012
40-49	13	3.08	(0.76)	549	3.11	(0.80)	545	3.01	(0.74)	1115	2.94	(0.73)	500	2.66	(0.73)	<0.001
50-59	12	2.50	(0.90)	424	2.97	(0.83)	471	2.87	(0.75)	1085	2.79	(0.76)	443	2.58	(0.76)	<0.001
60-69	22	2.14	(0.85)	555	2.80	(0.77)	659	2.81	(0.70)	1926	2.70	(0.73)	935	2.49	(0.71)	<0.001
70-79	24	2.29	(0.91)	265	2.47	(0.76)	320	2.61	(0.67)	831	2.63	(0.70)	382	2.37	(0.76)	<0.001
> 80	7	1.86	(0.69)	92	2.46	(0.72)	87	2.37	(0.72)	232	2.41	(0.74)	109	2.26	(0.71)	0.099

*p*-value is based on ANOVA. There was a significant effect of BMI for most age groups. Planned contrasts show that overweight and underweight significantly lowered the SRH, while the differences within the normal range for most groups did not show significant differences

The decline in SRH between age groups for those in the lowest weight category was most striking. SRH started out higher than for those in the normal range in all panels, but declined below all other BMI categories, and even passed obesity at around the 60 years age group. The pairwise comparisons between SRH levels for subjects with BMI <18.5 kg/m^2^ were significantly different from the overweight groups. The underweight group consequently had significant higher age-related decline before confounders such as time, comorbidity, mental health, and other risk factors were controlled for.

### Body weight and self-rated health with increasing age

**Fig. 3:**
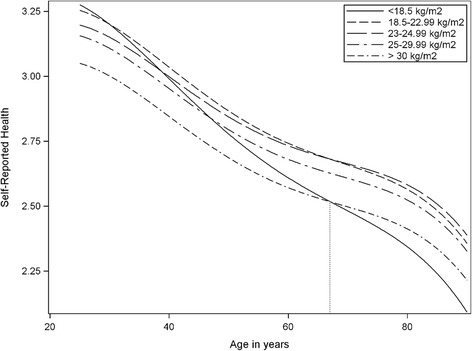
Self-reported health trajectories according to weight groups and age

Table 3 shows results of the complete case (CC) and inverse probability weighted (IPW) analyses. Figure 3 shows the results from the latent trajectory models and displays how SRH changed over time. Upper normal range (22-24.99 kg/m^2^) was our reference category. We see that SRH declined for all BMI groups with increasing age; however, the model also predicts different trajectories for different BMI levels. Having a BMI above 30 kg/m^2^ (−0.139, 95% CI: -0.257, −0.021) was never beneficial for SRH as the subjects grew older. For subjects with BMI below 18.5 kg/m^2^, we see a positive gap in SRH scores compared to the reference category (0.226, 95% CI: -0.094, 0.546); however, SRH interacted with age, which implies that it fell more rapidly as the subjects grew older (−0.059, 95% CI:-0.115, −0.002).

**Table 3:** Results from the complete case (CC) and inverse probability weighted (IPW) models with estimates for the association of subject-specific factors on Self-Reported Health trajectories

	CC			IPW		
	Coef.	95% CI.		Coef.	95% CI.	
Body mass index						
< 18.5 Kg/m^2^	0.238	−0.040	0.515	0.222	−0.091	0.536
18.5-22.99 Kg/m^2^	0.101	0.020	0.182	0.091	0.007	0.175
22-24.99 kg/m^2^	0.000	Reference		0.000	Reference	
25-29.99 kg/m^2^	−0.042	−0.118	0.035	−0.033	−0.112	0.045
> 30 kg/m^2^	−0.173	−0.283	−0.063	−0.137	−0.252	−0.021
BMI*age						
< 18.5 Kg/m^2^	−0.063	−0.112	−0.013	−0.058	−0.113	−0.002
18.5-22.99 Kg/m^2^	−0.015	−0.031	0.000	−0.014	−0.029	0.002
22-24.99 kg/m^2^	0.000	Reference		0.000	Reference	
25-29.99 kg/m^2^	−0.001	−0.015	0.013	−0.003	−0.017	0.011
> 30 kg/m^2^	0.002	−0.017	0.022	−0.004	−0.024	0.016
age (pr 10 years)	0.951	0.137	1.766	0.933	0.055	1.810
age^2^	−0.333	−0.572	−0.095	−0.341	−0.597	−0.085
age^3^	0.043	0.013	0.073	0.045	0.013	0.077
age^4^	−0.002	−0.003	−0.001	−0.002	−0.004	−0.001
Observation time (year)	−0.005	−0.006	−0.003	−0.004	−0.006	−0.003
Gender	−0.018	−0.034	−0.002	−0.010	−0.026	0.007
Specific medical conditions	−0.071	−0.075	−0.067	−0.063	−0.068	−0.059
Mental distress (standardized)	−0.222	−0.230	−0.215	−0.210	−0.218	−0.201
Resting heart rate (Standardized)	−0.040	−0.047	−0.032	−0.040	−0.049	−0.032
Systelic blood pressure (pr 10 mmHg)	0.003	−0.001	0.007	0.000	0.000	0.000
Total cholesterol	0.000	0.000	0.001	0.001	−0.005	0.008
Physical activity (4 levels)						
Inactive	0.000	Reference		0.000	Reference	
Light	0.102	0.085	0.119	0.091	0.073	0.108
Moderate	0.177	0.159	0.196	0.158	0.139	0.178
Intensive	0.212	0.187	0.237	0.192	0.165	0.218
Daily smoking (yes/no)	−0.092	−0.108	−0.076	−0.085	−0.102	−0.068
Constant	2.321	1.307	3.335	2.376	1.281	3.471
Random part						
Variance (constant)	0.111	0.105	0.117	0.165	0.158	0.171
Variance (Residual)	0.235	0.229	0.241	0.185	0.180	0.190

Complete case model (CC): Mixed-effects ML regression model, Wald chi2(23) =9184.16, Log likelihood = −25,102.758, *P* < 0.0001

Inverse probability weighted model (IPW): Mixed-effects ML regression model with inverse probability weighting for differential attrition, Wald chi2(23) =7683.26, Log likelihood = −29,943.958, *P* < 0.0001

When we calculated the differences at the maximum and minimum age of the data, we found that the confidence interval for underweight overlapped with the reference category (22-24.99 kg/m^2^) at age 25 (0.08, 95% CI: -0.02, 0.18), but it fell to −.30 (95% CI: −.19, −.41) below the reference category at age 90 in the fitted model. For obese subjects, the difference was −0.15 (95% CI: -0.05, −0.25) below the reference category at age 25 and −0.18 (95% CI: -0.07, −0.29) below at age 90. This implies that even though a low BMI was slightly beneficial at a young age, it represented an increasing risk with age, crossing the reference curve at age 38 and even crossing the obese trajectory at age 67 in the full fitted model.

### BMI and mortality risk

**Fig. 4:**
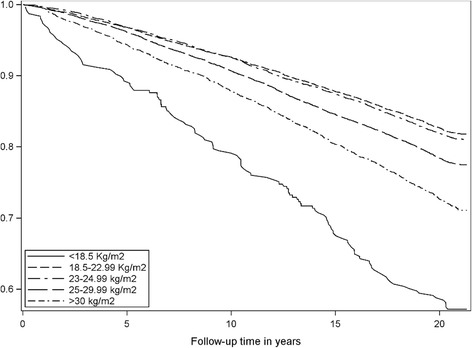
Kaplan-Meier survival function according to body mass index from 1994 to 2015

Table 4 shows the mortality rate and hazard ratio for BMI groups and SRH. The upper normal range (22-24.99 kg/m^2^) was our reference category. Subjects with BMI <18.5 Kg/m^2^ had twice the mortality rate (0.021) of the reference category (0.010) and also a higher rate than that of obese subjects (0.018). Figure 4 (Kaplan-Meier survival function) displays how the risk of death was most pronounced for the very thin but also increased gradually with increasing degrees of overweight. The Cox proportional hazard model shows that BMI <18.5 Kg/m^2^ was associated with 69% increased risk of all-cause death (HR 1.69, 95%CI: 1.38-2.06) and 18.5-22.99 Kg/m^2^ with a 14% increased risk (1.14, 95% CI: 1.05-1.25) as compared to the reference category (23-24.99 kg/m^2^). The HRs were controlled for age, gender, comorbidity, mental distress, systolic blood pressure, cholesterol, physical activity levels and daily smoking.

**Table 4:** Mortality rates and hazard ratio for all-cause mortality between categories of body mass index in the Tromsø study 1986-2008 until end of follow up on December 31, 2015

	Person Time (Years)	Died	Mortality rate	Hazard ratio ^a^	95% CI
Body mass index					
< 18.5 Kg/m^2^	5121	109	0.021	1.69	(1.38- 2.06)
18.5-22.99 Kg/m^2^	129,676	1122	0.009	1.14	(1.05- 1.25)
23-24.99 kg/m^2^ (ref)	107,253	1026	0.010	1.00	Reference
25-29.99 kg/m^2^	169,612	2160	0.013	0.97	(0.90- 1.05)
> 30 kg/m^2^	46,709	855	0.018	1.12	(1.02- 1.23)
Self-Reported Health					
Poor	10,762	416	0.039	2.54	(1.88- 3.45)
Not so good	117,369	2581	0.022	1.94	(1.55- 2.43)
Good	259,293	2045	0.008	1.30	(1.10- 1.54)
Very good (ref)	70,947	230	0.003	1.00	Reference
Time varying covariates					
Self-Reported Health				0.004	(1.00- 1.02)

^a^Hazard ratios (HR) are based on Cox proportional hazard and includes both SRH and BMI controlled for age, gender, comorbidity, mental health symptoms, systolic blood pressure, cholesterol, physical activity levels and daily smoking. For HRs shown in Table 4, is SRH time-varying but BMI time-invariant

When entering the updated BMI values measured at each consecutive wave into the model, we saw that the hazard ratios increased. This means that weight change increased the HRs. We built the models hierarchically, starting with BMI and then adding SRH and the other factors to the model. Controlled only for age and gender, the hazard ratio for underweight was 2.24 (95% CI: 1.84-2.73). When controlling for pathology and biometrics, the HR for the underweight subjects attenuated (HR 1.79, 95% CI: 1.46-2.18). Controlling for SRH alone reduced the HR to 2.05 (95% CI: 1.71-2.46). In the fully fitted model, we find that even though part of the risk was explained by comorbidity and physical condition, the subjects with the lowest BMI were still associated with a 69% higher mortality risk. An increasing HR, especially for the very thin, concurs with SRH being lower than the optimal BMI of 23-24.99 kg/m^2^ for the thin as age increases.

We investigated the interactions between gender, comorbidity, SRH and BMI, and found that obese men had a 25% higher risk (1.25, 95% CI: 1.04, 1.50) than obese women. SRH interacted with observation time, which implied that the hazard ratio increased with .8% for each year of observation. This is consistent with the findings in the latent trajectory model that show that SRH decreases with age.

## Discussion

This study shows that SRH changed with increasing age, but age affected the thinnest subjects more than all others. Underweight was associated with a higher mortality risk, and weight change increased this risk. Several authors argue that the health consequences of being underweight might be more severe in terms of premature mortality and quality of life, compared to being overweight [8–10, 16–18, 36–38]. Although being thin is an ideal, and despite the general advice for obese people to lose weight, our study indicates that being thin could in itself represent a health risk later in life. Inverse probability weighting (IPW) showed that loss to follow-up did not significantly affect this result, which supports the causal claim that being thin drives the later decline. It is interesting that normalizing the weight resulted in fewer comorbid conditions and lower mortality rates, and yet, lower SRH. SRH is strongly associated with biological factors, however, it is also known to be sensitive to factors such as coping skills, as well as mental distress, and social context [39]. One plausible explanation could be that general health advice is focused on losing weight, while normalizing the weight for underweight persons will imply gaining weight. This study cannot answer questions regarding health beliefs, but this could be addressed using qualitative methods.

Previous studies have shown that persons with BMI of <18.5 kg/m^2^ and ≥30 kg/m^2^ report impaired quality of life and SRH [40, 41]. Our study shows that this depends on age. Having a BMI above 30 kg/m^2^ was never beneficial for SRH as compared to the normal BMI range, while those with BMI below 18.5 kg/m^2^ started out with more beneficial levels but had a far more negative SRH trajectory as age increased. For underweight subjects, we see a positive gap in SRH scores compared to the other categories, but they fell more rapidly with age. Consequently, a low BMI was slightly beneficial at a young age, but it represented an increasingly negative factor with age, passing below the reference category at age 38 and even below the trajectory of obese subjects at age 67 in the fully fitted model.

Previous studies have shown that subjects who reported being obese in young adulthood only or in both young and middle adulthood experienced mortality rates that were 40%–90% higher than those subjects who were non-obese at either time [42]. Our study showed a similar association, but even higher rates for those who were very thin, with a 69% higher all-cause death risk (1.69, 95% CI: 1.38-2.06) as compared to only 12% for obese subjects (1.12, 95% CI: 1.02-1.23). The estimate is based on the baseline BMI at study entry. It indicates that starting as thin represented a mortality risk even though the body weight may change later in life, and although reaching a normal weight was beneficial for them.

The most underweight subjects were females among the youngest (<30 years) and oldest (>70 years) subjects. This is consistent with the difference we found between the group that started out as underweight and those who ended up as underweight. A Swedish study concluded that persons aged 75-90 who were overweight had a lower mortality risk than old persons with a BMI below 25 [43]. This is consistent with our findings for persons aged >67. Our study further shows that underweight in young age also represented a risk, even though underweight in younger adults is more likely to be associated with other medical issues (e.g. % fat mass), while in older adults underweight BMI could be an indicator of sarcopenia [44], malnutrition or other clinical conditions [6, 12].

Our models examined how body mass affected two different aspects of health. Mortality concerns survival, while SRH reflects the quality of survival. Being underweight affected both mortality and SRH negatively, while gaining weight had a negative association with all-cause mortality but might be beneficial for SRH. Weight loss is known to have a potential negative effect on health [13, 16–19, 21, 22]. Hence, it is reasonable to ask if the findings of our study are more a question of becoming, rather than being, underweight. The negative trajectory is explained as a combination of within-subject effects (i.e. becoming underweight) and between-subject effects (i.e. being underweight). It implies that changing BMI category (i.e. adding weight for those who were underweight) can affect SRH. However, when examining the effect of weight change in individual subjects, we find that weight gain was associated with lower SRH levels, even for those with a very low body mass. Although we would advise overweight persons to lose weight towards the normal range, gaining weight as general advice for underweight persons does not necessarily lead to better SRH.

Mortality was not affected by weight change in the same manner. The baseline BMI predicted a 69% increased risk, and when we modeled weight change by updating BMI values, we found that the risk increased to 79%, while for those in the lower normal range, the risk increased from 14% to 19%. This is consistent with a previous study of the Tromsø study cohort that showed that weight loss and gain were associated with increased all-cause mortality for men and in the subgroup of women who reported no weight-loss attempts [20].

Our study suggests that there is a healthy and an unhealthy underweight group, and that thinness due to waste is a risk. It seems plausible to distinguish between those who were initially underweight and those who became underweight (e.g. due to malnutrition). There are studies of nutrition and BMI status among older persons [6], but there are no studies that identify subgroups at risk among younger subjects. Our models control for known physical diseases, but not for eating disorders. Further studies should try to identify subgroups at particular risk among underweight persons. Health-related behavior explained 17% of the variance in our data, while gender and age (21%), comorbidity (23%) and mental distress (28%) are therefore important factors for an understanding of the decline. Our study suggests following up particularly gender differences, smoking status, the effect of weight change and development of comorbid diseases later in life.

We find more persons who exercise among the underweight subjects. This is consistent with being thin as part of a healthy life, but at the same time we find more persons who lead active lives and more non-smokers among the obese than we find from <25 kg/m^2^. It is timely to ask whether this might be an indication that health information is primarily targeting overweight subjects.

### Limitations

The Tromsø study was designed to represent a general population. Our latent trajectory model utilized 48.3% of TS 3, 45.3% of TS 4, 84.5% of TS 5 and 74.9% of TS 6. We used inverse probability weighting (IPW) to examine how the missing data affected the main finding of this study. IPW lowered the estimate for thin subjects and raised it for overweight subjects. The interaction with age attenuated, but the overall effect of age increased. The difference was that the underweight trajectory was equal in the CC and IPW models at age 25 (3.03) but ended 0.06 (3.5%) lower at age 90 in the weighted model. We see that loss to follow-up had a greater effect on the overweight (4.7%) and obesity groups (5.4%). The increased decline in the IPW model is consistent with the assumption that subjects well enough to participate several times are slightly healthier, but even so, loss to follow-up did not affect our overall results. Table 3 shows that confidence intervals (CIs) widened in the weighted model, but not substantially. No variable that was non-significant has become significant, or vice versa (except for gender and blood pressure that had CIs very close to zero). The findings thus remained basically unchanged by IPW, although there is an indication that the effect of ageing may be stronger in the general population than suggested by the CC analysis for under- and overweight subjects.

The survival analysis utilized the entire TS4 panel. Being able to use updated values reduces the bias in spite of missing data in the updated measurements. 51.3% of the participants attended only in TS4, 17.6% participated in all panels, while 31.1% reappeared in either TS5 or TS6. Using multiple imputation showed that missing data did not affect the estimates at the decimal level shown. We therefore conclude that the selection bias is within reasonable limits for both models.

It is of interest that the prevalence of underweight declined, but Tromsø 5-6 did not include participants <35 years and TS3 did not include participants >70 years, where we found most of the underweight subjects. No conclusions can therefore be drawn on the prevalence of underweight from this study. The small sample size of underweight individuals makes it difficult to fully analyze the causal claim of interest, but we can conclude that there are associations between underweight, self-reported health trajectories and mortality rates and that this relationship varies with age.

Although measured on an ordinal scale, the underlying phenomenon of SRH is continuous, and the scales represent similar logical increments. Furthermore, the distribution of SRH, apart from being staggered, resembled the shape of a normal distribution. Hence, an OLS regression model could be used for the analysis of independent associations in the multivariable model [45].

## Conclusion

BMI affected SRH and all-cause mortality independently from comorbidity, mental health, other biological risk factors or health-related behaviors. Being underweight was associated with excess mortality as compared to all other weight categories, and age affected the thinnest subjects more than all others. Weight increase was beneficial for mortality but not for SRH among the underweight. The rapid decline of SRH with increasing age suggests that particular attention should be paid to underweight subjects after 38 years of age.

## Additional file

Additional file 1: Table S1. Comparison of change in comorbitiy (HII) according to weight group at beginning and end of follow-up period. (PDF 60 kb)

## Abbreviations

BMI: Body mass index
CC: Complete case
IPW: Inverse probability weighting
SRH: Self-reported health
TS: The tromsø study

## References

[1] Tiggemann M, Cash TF, Smolak L (1980). Sociocultural perspectives on human appearance and body image. Body image: A handbook of science, practice, and prevention.

[2] World Health Organization (2000). The Asia-Pacific perspective. Redefining obesity and its treatment.

[3] World Health Organization (1995). Physical status. The use and interpretation of anthropometry. Report of a WHO expert committee. Technical Report Series, No 854.

[4] WHO Expert Consultation (2004). Appropriate body-mass index for Asian populations and its implications for policy and intervention strategies. London: Lancet.

[5] Dale C, Nüesch E, Prieto-Merino D, Choi M, Amuzu A, Ebrahim S (2015). Why do thin people have elevated all-cause mortality? Evidence on confounding and reverse causality in the association of adiposity and COPD from the British Women’s Heart and Health Study. PLoS One.

[6] Kvamme J-M, Olsen JA, Florholmen J, Jacobsen BK (2011). Risk of malnutrition and health-related quality of life in community-living elderly men and women: The Tromsø study. Qual Life Res.

[7] Kivimäki M, Shipley MJ, Bell JA, Brunner EJ, Batty GD, Singh-Manoux A (2016). Underweight as a risk factor for respiratory death in the Whitehall cohort study: exploring reverse causality using a 45-year follow-up. Thorax.

[8] Harpsøe MC, Nielsen NM, Friis-Møller N, Andersson M, Wohlfahrt J, Linneberg A, et al. Body mass index and risk of infections among women in the Danish National Birth Cohort. Am J Epidemiol. 2016; doi:10.1093/aje/kwv300. 10.1093/aje/kwv300 27188940

[9] Bucholz EM, Krumholz HA, Krumholz HM (2016). Underweight, markers of cachexia, and mortality in acute myocardial infarction: A prospective cohort study of elderly medicare beneficiaries. PLoS Med.

[10] Andersen KK, Olsen TS (2015). The obesity paradox in stroke: Lower mortality and lower risk of readmission for recurrent stroke in obese stroke patients. Int J Stroke.

[11] Kvamme J-M, Holmen J, Wilsgaard T, Florholmen J, Midthjell K, Jacobsen BK. Body mass index and mortality in elderly men and women: the Tromsø and HUNT studies. J Epidemiol Community Health. 2012;66:611-7. 10.1136/jech.2010.123232 PMC3368492 21321065

[12] Kvamme J-M, Wilsgaard T, Florholmen J, Jacobsen BK (2010). Body mass index and disease burden in elderly men and women: the Tromsø Study. Eur J Epidemiol.

[13] Huang K, Liu F, Han X, Huang C, Huang J, Gu D (2016). Association of BMI with total mortality and recurrent stroke among stroke patients: A meta-analysis of cohort studies. Atherosclerosis.

[14] Albergotti WG, Davis KS, Abberbock S, Bauman JE, Ohr J, Clump DA (2016). Association of pretreatment body mass index and survival in human papillomavirus positive oropharyngeal squamous cell carcinoma. Oral Oncol.

[15] Doleman B, Mills KT, Lim S, Zelhart MD, Gagliardi G (2016). Body mass index and colorectal cancer prognosis: a systematic review and meta-analysis. Tech Coloproctol.

[16] Warkentin LM, Das D, Majumdar SR, Johnson JA, Padwal RS (2014). The effect of weight loss on health-related quality of life: systematic review and meta-analysis of randomized trials. Obes Rev.

[17] Wagner DC, Short JL (2014). Longitudinal predictors of self-rated health and mortality in older adults. Prev Chronic Dis.

[18] Pan A, Kawachi I, Luo N, Manson JE, Willett WC, Hu FB, et al. Changes in body weight and health-related quality of life: 2 cohorts of US women. Am J Epidemiol. 2014; doi:10.1093/aje/kwu136. 10.1093/aje/kwu136 PMC4108043 24966215

[19] Maïano C, Normand CL, Aimé A, Bégarie J (2014). Lifestyle interventions targeting changes in body weight and composition among youth with an intellectual disability: A systematic review. Res Dev Disabil.

[20] Wilsgaard T, Jacobsen BK, Mathiesen EB, Njølstad I (2009). Weight loss and mortality: A gender-specific analysis of the Tromsø study. Gend Med.

[21] Newman AB, Yanez D, Harris T, Duxbury A, Enright PL, Fried LP (2001). Weight change in old age and its association with mortality. J Am Geriatr Soc.

[22] Alley DE, Metter EJ, Griswold ME, Harris TB, Simonsick EM, Longo DL (2010). Changes in weight at the end of life: characterizing weight loss by time to death in a cohort study of older men. Am J Epidemiol.

[23] Arcelus J, Mitchell AJ, Wales J, Nielsen S (2011). Mortality rates in patients with anorexia nervosa and other eating disorders A meta-analysis of 36 studies. Arch Gen Psychiatry.

[24] Ágh T, Kovács G, Supina D, Pawaskar M, Herman BK, Vokó Z (2016). A systematic review of the health-related quality of life and economic burdens of anorexia nervosa, bulimia nervosa, and binge eating disorder. Eat Weight Disord.

[25] Ganna A, Ingelsson E (2015). 5 year mortality predictors in 498 103 UK Biobank participants: a prospective population-based study. Lancet.

[26] Kaplan GA, Goldberg DE, Everson SA, Cohen RD, Salonen R, Tuomilehto J (1996). Perceived health status and morbidity and mortality: Evidence from the Kuopio Ischaemic Heart Disease Risk Factor Study. Int J Epidemiol.

[27] Desalvo KB, Jones TM, Peabody J, McDonald J, Fihn S, Fan V, et al. Health care expenditure prediction with a single item, self-rated health measure. Med Care. 2009;47 10.1097/MLR.0b013e318190b716. 10.1097/MLR.0b013e318190b716 19238099

[28] Jacobsen BK, Eggen AE, Mathiesen EB, Wilsgaard T, Njølstad I (2012). Cohort profile: The Tromsø Study. Int J Epidemiol.

[29] Rabe-Hesketh S, Skrondal A (2012). Multilevel and longitudinal modeling using stata, Volumes I and II.

[30] Pedersen A, Ellingsen C (2015). Data quality in the Causes of Death Registry. Tidsskr Nor Laegeforen.

[31] Lorem GF, Schirmer H, Emaus N. Health Impact Index. Development and validation of a method for classifying comorbid disease measured against self-reported health. PLoS One. 2016;11(2) 10.1371/journal.pone.0148830. 10.1371/journal.pone.0148830 PMC4746071 26849044

[32] Derogatis LR, Lipman RS, Rickels K, Uhlenhuth EH, Covi L (1974). The Hopkins Symptom Checklist (HSCL): A self-report symptom inventory. Behav Sci.

[33] Søgaard AJ, Bjelland I, Tell GS, Røysamb E (2003). A comparison of the CONOR Mental Health Index to the HSCL-10 and HADS. Nor Epidemiol.

[34] Wilsgaard T, Jacobsen BK, Arnesen E (2005). Determining lifestyle correlates of body mass index using multilevel analyses: The Tromsø Study, 1979–2001. Am J Epidemiol.

[35] Seaman SR, White IR (2013). Review of inverse probability weighting for dealing with missing data. Stat Methods Med Res.

[36] Pergialiotis V, Doumouchtsis SK, Perrea D, Vlachos GD (2016). The impact of underweight status on the prognosis of ovarian cancer patients: A meta-analysis. Nutr Cancer.

[37] Bruno A, Pace E, Cibella F, Chanez P. Body mass index and comorbidities in adult severe asthmatics. BioMed Res Int. 2014;2014:7. Article ID 607192. doi:10.1155/2014/607192. 10.1155/2014/607192 PMC4058470 24987694

[38] Wilsgaard T, Schirmer H, Arnesen E (2000). Impact of body weight on blood pressure with a focus on sex differences: The Tromsø Study, 1986-1995. Arch Intern Med.

[39] Mavaddat N, Kinmonth AL, Sanderson S, Surtees P, Bingham S, Khaw KT (2011). What determines Self-Rated Health (SRH)? A cross-sectional study of SF-36 health domains in the EPIC-Norfolk cohort. J Epidemiol Community Health.

[40] Ford ES, Moriarty DG, Zack MM, Mokdad AH, Chapman DP (2001). Self-reported body mass index and health-related quality of life: Findings from the Behavioral Risk Factor Surveillance System. Obes Res.

[41] Herman K, Hopman W, Rosenberg M (2013). Self-rated health and life satisfaction among Canadian adults: associations of perceived weight status versus BMI. Qual Life Res.

[42] Hirko KA, Kantor ED, Cohen SS, Blot WJ, Stampfer MJ, Signorello LB. Body mass index in young adulthood, obesity trajectory, and premature mortality. Am J Epidemiol. 2015; 10.1093/aje/kwv084. 10.1093/aje/kwv084 PMC4552269 25977515

[43] Dahl AK, Fauth EB, Ernsth-Bravell M, Hassing LB, Ram N, Gerstof D (2013). Body mass index, change in body mass index, and survival in old and very old persons. J Am Geriatr Soc.

[44] Legrand D, Vaes B, Matheï C, Swine C, Degryse J-M (2013). The prevalence of sarcopenia in very old individuals according to the European consensus definition: insights from the BELFRAIL study. Age Ageing.

[45] Hayes AF, Preacher KJ (2014). Statistical mediation analysis with a multicategorical independent variable. Br J Math Stat Psychol.

