# Supplementary material for: What is the impact of underweight on self-reported health trajectories and mortality rates: a cohort study
Source: Health Qual Life Outcomes. 2017 Oct 2;15:191. doi: 10.1186/s12955-017-0766-x (PMC5625617; doi:10.1186/s12955-017-0766-x)
Supplement: Additional file 1: Table S1. — Comparison of change in comorbitiy (HII) according to weight group at beginning and end of follow-up period. (PDF 60 kb) [file 12955_2017_766_MOESM1_ESM.pdf]

Additional file 1: Table S1. Comparison of change in comorbitiy (HII) according to weight group at beginning and end of follow-up period

	BMI group at the end of the study													
	<18.5 kg/m^2			18.5-24.99 kg/m^2			25-29.99 kg/m^2			>25 kg/m^2			P-value for SRH	P-value for HII
	N	SRH	HII	N	SRH	HII	N	SRH	HII	N	SRH	HII		
**BMI group at start of study**														
<18.5 kg/m^2	58	77.8 %	2.22	150	68.4 %	0.91	16	12.5 %	2.13	0			<0.001	0.006
18.5-24.99 kg/m^2	84	48.6 %	3.39	5536	69.9 %	1.59	3421	65.5 %	1.57	324	55.3 %	1.83	<0.001	<0.001
25-29.99 kg/m^2	4	0.0 %	0.00	316	52.5 %	2.10	2539	64.3 %	1.91	1365	52.3 %	2.30	<0.001	0.002
>30 kg/m^2	0			10	16.7 %	3.33	89	34.3 %	3.22	643	46.6 %	2.69	0.143	0.399

N= count, HII = Health impact index which measures comorbidity, BMI = Body mass index, SRH = % in good health.
